# Effect of a Brief Social Contact Video on Transphobia and Depression-Related Stigma Among Adolescents: A Randomized Clinical Trial

**DOI:** 10.1001/jamanetworkopen.2022.0376

**Published:** 2022-02-25

**Authors:** Doron Amsalem, Justin Halloran, Brent Penque, Jillian Celentano, Andrés Martin

**Affiliations:** 1 New York State Psychiatric Institute and Department of Psychiatry, Columbia University Vagelos College of Physicians and Surgeons, New York; 2 Child Study Center, Yale School of Medicine, New Haven, Connecticut; 3 Department of Social Work and Marriage and Family Therapy, Southern Connecticut State University, New Haven; 4 Simulated Participant Program, Teaching and Learning Center, Yale School of Medicine, New Haven, Connecticut

## Abstract

**Importance:**

Transphobia and stigma remain barriers to seeking mental health care for gender-diverse adolescents.

**Objective:**

To examine the utility of brief social contact–based video interventions of transgender protagonists with depression to reduce transphobia and depression-related stigma and increase treatment-seeking intentions among adolescents in the general population.

**Design, Setting, and Participants:**

During August 2021, a total of 1437 participants were recruited and randomly assigned to 1 of 4 video-based conditions in a 2:2:1:1 ratio: (1) transgender adolescent girls, (2) transgender adolescent boys, (3) cisgender adolescent girls, or (4) cisgender adolescent boys.

**Interventions:**

In each of the approximately 110-second videos, an empowered presenter shared their personal story about coping with depression and reaching out for help.

**Main Outcomes and Measures:**

The primary outcome was the score on the Attitudes Toward Transgender Men and Women (ATTMW) scale. Secondary outcomes were (1) a “gender thermometer” rating for warmth in transgender perception, (2) the Depression Stigma Scale (DSS) score, and (3) the General Health-Seeking Questionnaire (GHSQ) score.

**Results:**

Of the 1437 randomized participants, 1098 (76%) completed the postintervention assessment and passed all the validity tests (mean [SD] age, 16.9 [1.2] years; 481 [44%] male; 640 [58%] White). A significant change in attitudes toward transgender youth was found within the intervention group only (mean [SD] ATTMW scores: intervention group, 34.6 [23.1] at baseline to 32.8 [24.2] after intervention; *P* < .001; control group, 33.5 [23.4] at baseline to 32.4 [24.1] after intervention; *P* = .01). The mean (SD) total DSS scores decreased significantly across study groups (intervention: 1.3 [3.3]; control: 1.7 [3.3]; *P* < .001). A significant increase in intention to seek help from a parent was found in the intervention (mean [SD] GHSQ score, 0.2 [1.1]) and control (mean [SD] GHSQ score, 0.3 [1.2]) groups (*P* < .001), as was a decrease in those not wanting to seek help from anyone (mean [SD] GHSQ score: intervention, 0.2 [1.6], *P* = .009; control, 0.3 [1.2], *P* < .001) Secondary analyses revealed significant differences in baseline ATTMW scores and intervention effects between transgender and gender-diverse and cisgender participants and between lesbian, gay, bisexual, or queer (LGBQ) and straight participants (F = 36.7, *P* < .001) and heterosexual participants (F = 37.0, *P* < .001). A significant difference was also found in mean (SD) transgender warmth scores from baseline to after intervention between groups (2.6° [13.1°] in the intervention group vs 0.4° [8.3°] in the control group; *P* < .001).

**Conclusions and Relevance:**

In this randomized clinical trial, brief social contact–based videos proved efficacious in reducing transphobia and depression-related stigma and in increasing treatment-seeking intentions among adolescents in the general population. By personifying, individualizing, and providing face and voice to the experience of transgender youth, other adolescents, especially those who are cisgender and/or of a heterosexual orientation, can gain empathetic insights into the lives of their often marginalized and stigmatized fellow youth.

**Trial Registration:**

ClinicalTrials.gov Identifier: NCT04969003

## Key Points

**Question:**

Can a 110-second video of a transgender protagonist describing their personal history of coping with depression reduce adolescent transphobia and depression-related stigma?

**Findings:**

In this randomized clinical trial of 1098 adolescents, a significant change in attitudes was found toward transgender youth only in the intervention groups, especially among participants who self-identified as cisgender and/or of heterosexual orientation. As anticipated, a significant reduction in depression-related stigma was also found across all study groups.

**Meaning:**

Brief social contact–based videos proved efficacious in reducing adolescent transphobia and depression-related stigma.

## Teaser

This randomized clinical trial examines whether video interventions of transgender adolescents discussing their experience with depression reduce transphobia and depression-related stigma and increase treatment seeking among gender-diverse adolescents.

## Introduction

Transgender and gender-diverse (TGD) youth are disproportionally affected by depression, anxiety, and suicidal ideation when compared with their cisgender peers.^1,2,3,4,5^ Although gender diversity by itself is not pathological and does not require mental health treatment, mental health issues can be secondary to gender dysphoria, and TGD youth may benefit from mental health treatment and gender-affirming medical care. The prevalence of suicidal ideation among TGD youth has been reported to be almost two-thirds in some studies,^6,7,8,9^ with an alarming 41% suicide attempt rate. Data indicate that the proportion of youth who openly self-identify as TGD has increased substantially over the years.^10,11^ The most recent Centers for Disease Control and Prevention Youth Risk Behavior Survey^12^ reported that 1.8% of high school youth identify as transgender, and recent studies^13,14,15^ among 3441 young people have found that 90 (3%) identified as gender nonbinary.

The stigma surrounding mental illness acts as a barrier to young people seeking care, such that reducing stigmatized perceptions among young people could enhance their likelihood to seek help or treatment.^16,17,18,19,20,21^ It is well known that transphobia, a form of stigma against TGD youth, may lead to social discrimination, minority stress, and internalized self-hate, creating risk factors for mental illness in this population.^22^ For example, transgender high school students report significantly higher rates of victimization and harassment than their cisgender peers and are more likely to feel unsafe.^23^ Considered through the minority stress model, transgender youth struggle with distal factors, such as discrimination, and proximal ones, such as concealment and internalized transphobia.^24^ Moreover, recent changes in legislation, such as bills that target gender-affirming medical care or place restrictions on bathrooms or sports for transgender youth, are increasing.^25,26^ Thus, TGD youth struggling with depression often face the dual stigma of marginalized gender identity and mental illness.

Social contact–based interventions are the most successful way to reduce stigma.^27^ Video-based social contact interventions have effectively improved attitudes toward mental illness and reduced stigma and discrimination.^28,29^ A previous study^15^ among 1183 adolescents demonstrated the efficacy of brief videos (102-113 seconds each) in decreasing depression-related stigma and increasing participants’ reported willingness to seek mental health care. Brevity has advantages, including lower cost, less resource use, and greater ease of dissemination to large audiences. Shorter videos are also better suited to younger audiences. Social contact with TGD individuals has been shown to improve attitudes and reduce transphobia, but no study to date has examined the efficacy of a brief video intervention in changing the perceptions of general-population youth toward TGD people.^30,31,32,33,34^

With these considerations in mind, we conducted a randomized clinical trial of adolescents in the general population to test the utility of a brief video-based intervention of transgender adolescent protagonists in order to reduce transgender-related stigma (transphobia) and depression-related stigma and increase treatment-seeking intentions. We hypothesized that when compared with the control condition of cisgender protagonists describing their depression and pathways to care, the transgender protagonist interventions would result in greater reduction in transphobia and similar changes in depression-related stigma and treatment-seeking intentions.

## Methods

### Participants and Recruitment

We recruited 1437 evaluable participants using CloudResearch,^35^ a crowdsourcing platform widely used in behavioral research and with extensive experience in recruiting underrepresented groups, including minors. We included only English-speaking youth, 14 to 18 years of age, living in the US. We focused on this age range because it overlaps with the median age of onset of major depressive disorder and/or suicidal ideation. All participants reviewed an informed assent page and provided written informed consent (parental consent was waived). Participants were recruited and completed the study during August 2021. All data were deidentified. This randomized clinical trial was approved by the Yale Human Investigations Committee and followed the Consolidated Standards of Reporting Trials (CONSORT) reporting guideline. The trial protocol is available in Supplement 1.

**Figure 1.**
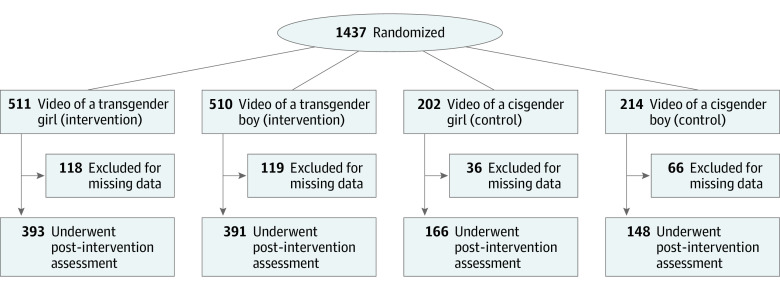
Study Flow Diagram

We randomly assigned participants to 1 of 4 video conditions on a 2:2:1:1 ratio (transgender girl, transgender boy, cisgender girl, and cisgender boy) (Figure 1). For participants who self-identified as transgender or gender nonbinary (n = 131), we constrained randomization to the transgender girl or transgender boy conditions. There are 2 explanations for this decision. First, an earlier study^15^ showed a significant effect for the intervention groups in reducing depression-related stigma and increasing treatment-seeking intent, especially among viewers who shared demographic characteristics with the protagonist. Considering the relatively high level of depression and suicidality among TGD adolescents, randomizing those teens to 1 of the intervention groups seemed clinically justified for this high-risk population. Second, based on the previous findings^15^ that greater identification with the video presenter correlates with greater effect, we were interested in examining whether a transgender person watching someone who identifies similarly would show a greater decrease in stigma and increase in treatment seeking. We would not anticipate a change in transphobia among TGD adolescents exposed to cisgender protagonists. Assuming a low number of participants who would identify as TGD, we only assigned those individuals into 1 of 2 intervention groups.

We used several accepted methods to exclude invalid participants to ensure the quality of the collected data, as described in a previous study.^15^ In addition, we used 3 questions to exclude inattentive or disengaged participants, each phrased in a consistent way and requiring a single, forced answer (eg, “Please mark the third option below”). Volunteers were compensated $3.50 for their participation. We directed respondents who agreed to participate to an online data collection platform (Qualtrics).

### Intervention

We used 2 brief intervention videos (114-118 seconds each), excerpted from filmed interviews with two 17-year-old transgender adolescents, 1 female (“Monica”) and 1 male (“Parker”). One of us (J.C.), a transgender woman with experience working with gender-fluid youth, helped develop the scripts and supported the actors during the rehearsal and filming sessions. These scripts aimed to reflect some of the objective realities of being transgender in a way that was true to life, while avoiding exaggeration that could further entrench gender stereotypes. For example, the youth described their difficulties of “living in the wrong body” and feeling “trapped and [as if] there was no way out.” They also explained that because of these sentiments their depression worsened and led to thoughts that life is not worth living and even of dying by suicide. Later, they discussed how sharing these intimate feelings with family and friends and subsequently receiving professional help changed their lives for the better. The control videos (102-113 seconds each), the same ones used in the previous study,^15^ included 2 young cisgender adolescent protagonists, 1 a female (“Ali”) and 1 a male (“Danny”), who described how they had coped with depressive symptoms and ultimately recovered through the help and support they received. The 4 video clips are available for viewing through links in eAppendix 1 in Supplement 2.

### Instruments

The primary outcome was the Attitudes Toward Transgender Men and Women (ATTMW) scale, a measure of transphobic attitudes toward transgender individuals.^36^ We adjusted the wording of the scale to assess the attitudes toward transgender adolescents and combined the female and male subscales into a single, overarching summary scale. The scale is scored along a 7-point Likert-type range that goes from strongly disagree (score of 1) to strongly agree (score of 7). Higher scores indicate greater transphobia. The ATTMW is highly reliable and has a Cronbach α of 0.97.^36^

Secondary outcome measures included a “gender thermometer,”^37^ a tool developed to assess attitudes regarding sexual orientation and gender diversity, to gauge participants’ attitudes around gender diversity. The thermometer provides the following prompt: “Using a scale from 0 to 100, please tell us about your personal feelings toward each of the following groups of friends, teachers, or colleagues. As you do this task, think of an imaginary thermometer. The warmer or more favorable you feel toward the group, the higher the number you should give it. The colder or less favorable you feel, the lower the number. If you feel neither warm nor cold toward the group, rate it 50.” We asked respondents about their attitudes toward (1) heterosexual; (2) lesbian, gay, bisexual, and queer (LGBQ); and (3) transgender people. Higher ratings indicate warmer, closer, more favorable feelings toward the group in question, whereas lower ratings indicate colder, more distant, or negative feelings. As in the previous study,^15^ we assessed stigma toward depression using the Depression Stigma Scale (DSS)^38^ and treatment-seeking intentions using the General Help-Seeking Questionnaire (GHSQ).^39^

### Statistical Analysis

We used Pearson χ^2^ and 1-way analysis of variance (ANOVA) to compare demographic variables across groups. We used paired 2-tailed *t* tests to compare ATTMW and “temperature” mean scores at baseline and after intervention. We also used 1-way ANOVA to compare baseline differences in outcome measures across gender and sexual orientation subgroups. We then used paired *t* tests to compare changes between the baseline and postintervention periods across study groups. For all *t* tests, we used the Bonferroni correction, considering as significant only those results with *P* < .001. We conducted all statistical analyses using SPSS software, version 26.0 (IBM Inc).

## Results

### Sample Characteristics

We recruited and proportionally randomized 1437 participants, of whom 1098 (76%) completed the postintervention assessment and passed all the validity tests (mean [SD] age, 16.9 [1.2] years; 473 [43%] female; 481 [44%] male; 131 [12%] transgender or nonbinary; 183 [17%] African American or Black; 89 [8%] Asian; 261 [24%] Hispanic or Latinx; 28 [3%] American Indian or Alaska Native; 640 [58%] White; and 158 [14%] of other race or ethnicity, including 2 Middle Eastern, 126 unspecified, and 30 who preferred not to answer) (Figure 1). Demographic characteristics did not differ between those who completed the study and those who dropped out (n = 339). Study groups did not differ by gender, age, race, or ethnicity, but LGBQ participants were underrepresented in the control groups because TGD participants were preferentially randomized to the intervention groups (Table 1).

**Table 1.** Demographic Characteristics of Study Participants^a^

Characteristic	Intervention (transgender protagonist)		Control (cisgender protagonist)		Total (N = 1098)	Statistic	*P* value
	Female (n = 393)	Male (n = 391)	Female (n = 148)	Male (n = 166)			
Age, mean (SD), y	16.9 (1.1)	16.8 (1.2)	17.1 (1.1)	16.8 (1.2)	16.9 (1.2)	2.07^b^	.10
Gender							
Female	163 (42)	154 (39)	70 (47)	86 (52)	473 (43)	1.39^c^	.71
Male	155 (39)	168 (43)	78 (53)	80 (48)	481 (44)		
Nonbinary	45 (12)	46 (12)	NA	NA	91 (8)		
Transgender	27 (7)	13 (3)	NA	NA	40 (4)		
Prefer not to answer	3 (1)	10 (3)	NA	NA	13 (1)		
Sexual orientation							
Heterosexual	222 (57)	219 (56)	107 (72)	114 (69)	662 (60)	27.1^c^	.001
LGBQ	135 (34)	119 (30)	33 (22)	36 (22)	323 (29)		
I am not sure	30 (8)	38 (10)	7 (5)	12 (7)	87 (8)		
Prefer not to answer	6 (2)	15 (4)	1 (1)	4 (2)	26 (2)		
Race and ethnicity							
African American or Black	61 (16)	75 (19)	18 (12)	29 (18)	183 (17)	12.0^c^	.44
Asian	37 (9)	28 (7)	14 (10)	10 (6)	89 (8)		
Hispanic or Latinx	96 (24)	87 (22)	38 (26)	40 (22)	261 (24)		
American Indian or Alaska Native	6 (2)	11 (3)	5 (3)	6 (4)	28 (3)		
White	227 (58	229 (59)	85 (57)	99 (60)	640 (58)		
Other^d^	62 (16)	48 (12)	26 (18)	22 (13)	158 (14)		

Abbreviations: LGBQ, lesbian, gay, bisexual, or queer; NA, not applicable.

^a^ Data are presented as number (percentage) of study participants unless otherwise indicated.

^b^ One-way analysis of variance.

^c^ Pearson χ^2^ test.

^d^ Other includes 126 with unspecified race, 30 who preferred not to answer, and 2 of Middle Eastern race.

### Attitudes Toward Transgender Men and Women 

Although we found no between-group difference before and after the intervention (mean [SD] ATTMW scores of 1.8 [7.5] in the intervention group vs 1.1 [8.5] in the control group; independent *t* = 1.1, *df* = 879, *P* = .25), a difference was found within the intervention group only vs the control group (34.6 [23.1] at baseline to 32.8 [24.2] at after intervention [paired *t* = 5.3, *P* < .001] vs 33.5 [23.4] at baseline to 32.4 [24.1] after intervention [paired *t* = 2.6, *P* = .01]). The intervention group had significant reductions in 6 of 12 ATTMW items, and the control group in only 1 (Table 2). We found relatively low baseline mean (SD) ATTMW scores for TGD people (14.5 [5.9]) (ie, low stigmatization). One-way ANOVA showed a significant difference between mean (SD) baseline scores for boys (28.5 [27.4]), girls (17.9 [21.0]), and TGD adolescents (12.9 [7.2]; F = 36.7, *P* < .001). One-way ANOVA also showed a differential baseline pattern between heterosexual (27.9 [27.3]) and LGBQ adolescents (12.4 [11.9]; F = 37.0, *P* < .001).

**Table 2.** Comparison Between Video Intervention and Control Group Scores on the Attitudes Toward Transgender Men and Women Scale (ATTMW) Among Cisgender Participants^a^

Attitude toward transgender adolescents	Intervention (transgender protagonist) (n = 640)				Control (cisgender protagonist) (n = 314)			
	Mean (SD)		*t* ^b^	*P* value	Mean (SD)		*t* ^b^	*P* value
	Baseline	Post			Baseline	Post		
1. Will never really be women/men	3.0 (2.1)	2.8 (2.1)	3.1	<.001	3.0 (2.2)	2.9 (2.1)	1.3	.18
2. Are not really females/males	3.0 (2.1)	2.9 (2.2)	2.6	.011	2.9 (2.1)	2.9 (2.1)	0.4	.68
3 Will only be able to look like women/men, but not be women/men	3.0 (2.1)	2.8 (2.1)	3.2	<.001	3.0 (2.1)	3.0 (2.1)	0.8	.40
4. Are unable to accept who they really are	3.0 (2.1)	2.9 (2.1)	1.8	.07	3.0 (2.1)	2.9 (2.0)	2.2	.03
5. Are trying to be someone they’re not	2.9 (2.1)	2.8 (2.1)	2.2	.03	2.9 (2.2)	2.9 (2.1)	0.1	.91
6. Are denying their DNA	3.3 (2.2)	3.1 (2.2)	2.9	.003	3.3 (2.2)	3.1 (2.2)	2.9	.004
7. Cannot just “identify” as females/males	3.0 (2.1)	2.9 (2.1)	2.3	.019	3.1 (2.2)	2.9 (2.1)	2.9	.005
8. Are unnatural	3.1 (2.2)	2.9 (2.1)	3.1	<.001	2.9 (2.1)	2.9 (2.1)	0.1	.91
9. Don’t really understand what it means to be a female/male	3.0 (2.0)	3.0 (2.1)	0.1	.97	2.9 (2.0)	2.9 (2.1)	0.6	.50
10. Only think they are females/males	3.3 (2.0)	3.0 (2.1)	4.8	<.001	3.1 (1.9)	3.0 (2.0)	2.4	.02
11. Are defying nature	3.2 (2.1)	3.0 (2.1)	3.8	<.001	3.1 (2.0)	3.0 (2.0)	1.6	.11
12. There is something unique about being a woman/man that transgender adolescents can never experience	3.7 (2.1)	3.3 (2.1)	6.9	<.001	3.5 (2.1)	3.3 (2.1)	3.4	<.001
Total scores	37.3 (21.8)	35.4 (23.3)	5.3	<.001	36.7 (22.0)	35.5 (23.0)	2.6	.01

^a^ Item ratings ranged from 1 (strongly disagree) to 7 (strongly agree) on a Likert-type scale, with higher scores indicating higher stigma. Cohen *d* effect sizes ranged from 0.13 to 0.27.

^b^ Paired *t* test.

**Figure 2.**
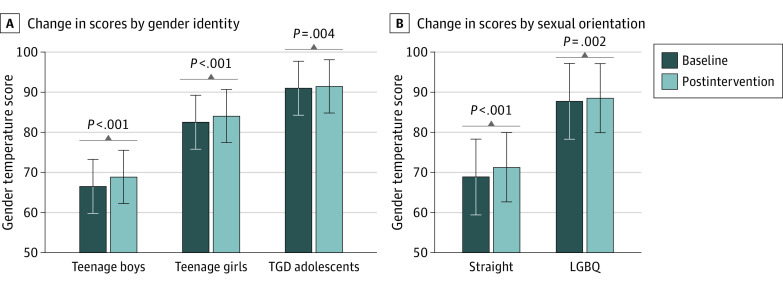
Comparison of Baseline and Postintervention Scores on Personal Feelings Toward Transgender People After Social-Contact Video Intervention A higher score indicates improvement in stigmatizing attitudes and warmer feeling toward transgender people. Error bars indicate standard error of the mean. The Cohen *d* effect sizes ranged from 0.22 to 0.25. LGBQ indicates lesbian, gay, bisexual, and queer; TGD, transgender and gender diverse.

Independent *t* tests showed a significant difference between the changes in “gender temperature” mean (SD) scores from baseline to after intervention in the intervention vs control groups (2.6° [13.1°] vs 0.4° [8.3°]; *t* = 3.2, *P* < .001). Paired *t* tests showed a significant difference in the intervention groups only (70.5° [32.3°] at baseline to 73.0° [32.0°] after intervention, *t* = 5.5, *P* < .001, Cohen *d* = 0.20 in the intervention group vs 69.3° [32.8°] at baseline to 69.8° [32.4] after intervention, *P* = .36, in the control group). One-way ANOVA showed a significant difference between mean (SD) baseline “gender temperature” ratings between boys (54.6° [34.7°]), girls (78.4° [26.9°]), and TGD adolescents (91.0° [15.5°]; F = 92.4, *P* < .001) (Figure 2A). One-way ANOVA also showed a differential response pattern in baseline “gender temperature” ratings between heterosexual (58.6° [34.8°]) and LGBQ (87.3° [19.0°]; F = 57.4, *P* < .001) participants (Figure 2B). Independent *t* tests showed a significant difference in the change from baseline to after intervention between the heterosexual (3.7° [16.4°]) and LGBQ groups (1.2° [5.9°]; F = 36.4, *P* < .001, Cohen *d* = 0.19).

### Depression Stigma Scale 

As hypothesized, all study groups demonstrated a significant change between preintervention and postintervention DSS scores, and univariate ANOVA showed no between-group differences. Table 3 presents the 9 DSS item mean scores and compares baseline and postintervention ratings between the intervention (n = 784) and control (n = 314) groups. The mean (SD) DSS total scores decreased significantly across study groups (intervention: 1.3 [3.3]; control: 1.7 [3.3]; paired *t* ≥ 9.4; *P* < .001; Cohen *d* ≥ 0.38). We found significant reductions in the same 4 (of 9) items across study groups: *weak* (1.8 [1.1] to 1.7 [1.1], *t* = 3.7, *P* < .001 in the intervention group vs 1.9 [1.2] to 1.7 [1.1], *t* = 4.7, *P* < .001 in the control group), *dangerous* (2.2 [1.1] to 1.9 [1.1], *t* = 6.9, *P* < .001 in the intervention group to 2.3 [1.1] to 1.9 [1.1], *t* = 7.6, *P* < .001 in the control group), *unpredictable* (2.9 [1.2] vs 2.6 [1.2], *t* = 8.8, *P* < .001 in the intervention group vs 2.9 [1.1] to 2.5 [1.2], *t* = 6.7, *P* < .001 in the control group), and *wouldn’t tell* (3.0 [1.3] to 2.5 [1.3], *t* = 9.4, *P* < .001 in the intervention group vs 3.0 [1.3] to 2.5 [1.3], *t* = 7.4, *P* < .001 in the control group).

**Table 3.** Comparison Between Social-Based Video Intervention and Control Group Scores on the Depression Stigma Scale (DSS)^a^

Attitude toward depression	Intervention (transgender protagonist) (n = 784)				Control (cisgender protagonist) (n = 314)			
	Mean (SD)		*t* ^b^	*P* value	Mean (SD)		*t* ^b^	*P* value
	Baseline	Post			Baseline	Post		
1. People with depression could snap out of it if they wanted	1.7 (1.1)	1.7 (1.1)	2.0	.04	1.8 (1.2)	1.7 (1.2)	1.5	.13
2. Depression is a sign of personal weakness	1.8 (1.1)	1.7 (1.1)	3.7	<.001	1.9 (1.2)	1.7 (1.1)	4.7	<.001
3. Depression is not a real medical illness	1.4 (0.9)	1.4 (0.8)	0.5	.59	1.5 (0.9)	1.4 (0.9)	1.7	.09
4. People with depression are dangerous	2.2 (1.1)	1.9 (1.1)	6.9	<.001	2.3 (1.1)	1.9 (1.0)	7.6	<.001
5. It is best to avoid people with depression, so you don’t become depressed yourself	1.6 (0.9)	1.5 (0.9)	2.6	.009	1.6 (0.9)	1.5 (0.8)	3.2	.002
6. People with depression are unpredictable	2.9 (1.2)	2.6 (1.2)	8.8	<.001	2.9 (1.1)	2.5 (1.2)	6.7	<.001
7. If I had depression, I would not tell anyone	3.0 (1.3)	2.6 (1.3)	9.4	<.001	3.0 (1.3)	2.5 (1.3)	7.4	<.001
8. I would not employ someone if I knew they had been depressed	1.6 (1.0)	1.5 (0.9)	2.4	.02	1.6 (0.9)	1.5 (0.9)	1.6	.10
9. I would not vote for a politician if I knew they had been depressed	1.7 (1.0)	1.7 (1.0)	0.2	.87	1.6 (1.0)	1.7 (1.0)	0.9	.33
Total scores	17.8 (5.5)	16.5 (6.2)	10.6	<.001	18.2 (5.4)	16.4 (5.9)	9.4	<.001

^a^ Item ratings ranged from 1 (strongly disagree) to 5 (strongly agree) on a Likert-type scale, with higher scores indicating higher stigma. Cohen *d* effect sizes ranged from 0.13 to 0.53.

^b^ Paired *t* test.

### General Help-Seeking Questionnaire 

We found a significant increase in intention to seek help from a parent in the intervention (mean [SD] GHSQ score, 0.2 [1.1]) and control (mean [SD] GHSQ score, 0.3 [1.2]) groups (paired *t* ≥ 3.5, *P* < .001) and a decrease in those not wanting to seek help from anyone (mean [SD] GHSQ score, 0.2 [1.6], *t* = 2.6, *P* = .009 in the invention group vs 0.3 [1.2], *t* = 3.9, *P* < .001 in the control group) (eAppendix 2 in Supplement 2).

## Discussion

In this randomized clinical trial, we tested the efficacy of 2 brief social contact–based video interventions that featured 2 transgender adolescents aiming to reduce transphobia and depression-related stigma and to increase treatment-seeking intentions among 1098 adolescents in the general population. As hypothesized, these approximately 110-second videos had a significantly greater effect on lowering transphobia compared with 2 comparable videos that featured cisgender adolescents and a similar effect in changing depression-related stigma and treatment-seeking intentions. The latter finding is a replication of a previous study^15^ among 1183 adolescents. Each of the 4 videos provided direct and personal exposure to the struggles and difficulties of a transgender or cisgender protagonist, who presented as a potential peer. Consistent with previous studies^13,14,15^ conducted among young adults, this randomized clinical trial showed the effectiveness of a brief contact-based video intervention in changing stigmatized perceptions and attitudes among adolescents in the general population. Our data show the effect of an empowered presenter with personal lived experience, seen as a potential peer, and with emotional characteristics that resonate with the audience. Their brief video depictions disconfirmed stereotypes by balancing difficulties with messages of hope.^40^

A previous study^41^ of interventions to reduce transphobia have a variety of contexts, as specifically designed to create changes at the structural, interpersonal, or individual levels. Existing studies on social contact–based interventions are scarce^42^ and have included transgender speaker panels,^22^ game-based interventions,^43^ and film-based interventions to improve parents’ responses to their LGBTQ children.^44^ Brief social contact interventions may better suit younger audiences who are used to consuming knowledge through social media platforms (for example, Instagram and TikTok limit the length of uploaded videos to 1 minute). In addition, we can anticipate the scalability and replicability of the brief video intervention approach given low production costs and ease of adjusting underlying scripts, target populations, and specific goals.

Our findings regarding baseline differences across gender and sexual orientation in attitudes toward transgender youth are consistent with previous studies.^32,45,46,47^ However, in secondary, post hoc analyses, we found that the intervention had the greatest effect among male and/or heterosexual adolescents and the smallest effect among participants who self-identified as TGD and/or LGBQ. This finding contradicts previous data,^13,48^ which showed a positive correlation between the intervention effect and the level of identification with the video protagonist. One possible explanation for this unexpected finding lies within the differences at baseline: men and/or heterosexual participants had a higher rate of stigma at baseline, and TGD and/or LGBQ participants had more favorable attitudes, introducing a possible ceiling effect that limited further improvement.

Taken together, these findings have several implications. The first and more clinically relevant is that short videos hold promise as interventions that can have a substantial public health effect. The findings from this report corroborate those from our earlier studies^13,14,15^: a brief social-based video intervention reduces depression-related stigma and increases treatment-seeking intentions. The second implication is regarding the interventions specifically tailored to transgender youth. By personifying, individualizing, and providing face and voice to the experience of transgender youth, other adolescents, especially those of cisgender and/or heterosexual orientation groups, can gain empathetic insights into the lives of their often marginalized and stigmatized peers.^49,50^ Third, these interventions can be used for educational purposes in general, specifically in training health care professionals. A previous study^51^ has shown that didactic information is insufficient to improve medical students’ perspectives toward transgender people.

### Limitations

Our study has several limitations. First, results on the ATTMW, our primary outcome measure, were equivocal, with strong within-group findings (intervention greater than control) but no between-group difference (intervention equal to control). “Gender temperature” ratings, measuring a similar construct, were significant both within and between groups. This unexpected finding may be due to possible confounding between depressive symptoms and protagonists’ descriptions of their earlier gender dysphoria. Future studies would benefit from disambiguating these 2 factors because they each have a strong emotional pull on participants and may have obscured findings. Second, findings may be limited to CloudResearch participants, who may not be fully representative of the general population. Fifty-eight percent of participants described their race as White, 14% as African American, and 8% as Asian, and 24% reported Hispanic ethnicity, slightly diverging from the US population’s distribution. Third, we did not use standard criterion to collect gender identity. Current guidelines suggest the 2-step method, in which the first question addresses the participant’s sex assigned at birth and the second their gender identity. Fourth, by randomly assigning transgender or nonbinary individuals to only 2 of the 4 intervention videos, we may have obscured response patterns unique to this sizable fraction of our sample (12%). Fifth, our study included only 2 time points and did not evaluate longer-term effects. However, we are not aware of any such studies with underage participants. Sixth, we only assessed attitudes, the reporting of which is subject to social desirability and may not be indicative of actual behavior. However, a meta-analysis^52^ of the experimental evidence available by 2006 showed that change in attitudes does in fact lead to behavioral change.

## Conclusions

In this randomized clinical trial, a brief contact-based video intervention effectively reduced reported attitudes of transphobia, particularly among cisgender and/or heterosexual youth. It also reduced depression-related stigma and increased treatment-seeking intentions among adolescents in the general population. This simple, easy-to-disseminate online intervention may have the added potential of improving access to treatment specifically among TGD adolescents with depression or suicidal thoughts. Future studies should explore whether and how to tailor brief contact-based interventions to specific populations and to emerging online platforms for content dissemination.

## Notes

Supplement 1.
Trial Protocol

Supplement 2. **eAppendix 1.** Links to Four Video Conditions **eAppendix 2.** Comparison Between Baseline and Postintervention Scores (n = 1,009) on the General Help-Seeking Questionnaire (GHSQ)

Supplement 3.
Data Sharing Statement

## References

[1] Price-Feeney M, Green AE, Dorison S. Understanding the mental health of transgender and nonbinary youth. J Adolesc Health. 2020;66(6):684-690. doi:10.1016/j.jadohealth.2019.11.314 31992489

[2] Connolly MD, Zervos MJ, Barone CJ II, Johnson CC, Joseph CLM. The mental health of transgender youth: advances in understanding. J Adolesc Health. 2016;59(5):489-495. doi:10.1016/j.jadohealth.2016.06.012 27544457

[3] Reisner SL, Vetters R, Leclerc M, . Mental health of transgender youth in care at an adolescent urban community health center: a matched retrospective cohort study. J Adolesc Health. 2015;56(3):274-279. doi:10.1016/j.jadohealth.2014.10.264 25577670 PMC4339405

[4] Becerra-Culqui TA, Liu Y, Nash R, . Mental health of transgender and gender nonconforming youth compared with their peers. Pediatrics. 2018;141(5):e20173845. doi:10.1542/peds.2017-3845 29661941 PMC5914494

[5] Costa R, Dunsford M, Skagerberg E, Holt V, Carmichael P, Colizzi M. Psychological support, puberty suppression, and psychosocial functioning in adolescents with gender dysphoria. J Sex Med. 2015;12(11):2206-2214. doi:10.1111/jsm.13034 26556015

[6] Eisenberg ME, Gower AL, McMorris BJ, Rider GN, Shea G, Coleman E. Risk and protective factors in the lives of transgender/gender nonconforming adolescents. J Adolesc Health. 2017;61(4):521-526. doi:10.1016/j.jadohealth.2017.04.014 28736148 PMC5626022

[7] Thoma BC, Salk RH, Choukas-Bradley S, Goldstein TR, Levine MD, Marshal MP. Suicidality disparities between transgender and cisgender adolescents. Pediatrics. 2019;144(5):e20191183. doi:10.1542/peds.2019-1183 31611339 PMC7011156

[8] Toomey RB, Syvertsen AK, Shramko M. Transgender adolescent suicide behavior. Pediatrics. 2018;142(4):e20174218. doi:10.1542/peds.2017-4218 30206149 PMC6317573

[9] The Trevor Project. 2020 national survey on LGBTQ youth mental health. 2020. Accessed April 26, 2021. https://www.thetrevorproject.org/wp-content/uploads/2020/07/The-Trevor-Project-National-Survey-Results-2020.pdf

[10] Meerwijk EL, Sevelius JM. Transgender population size in the united states: a meta-regression of population-based probability samples. Am J Public Health. 2017;107(2):e1-e8. doi:10.2105/AJPH.2016.303578 28075632 PMC5227946

[11] Richards C, Bouman WP, Seal L, Barker MJ, Nieder TO, T’Sjoen G. Non-binary or genderqueer genders. Int Rev Psychiatry. 2016;28(1):95-102. doi:10.3109/09540261.2015.1106446 26753630

[12] Johns MM, Lowry R, Andrzejewski J, . Transgender identity and experiences of violence victimization, substance use, suicide risk, and sexual risk behaviors among high school students - 19 states and large urban school districts, 2017. MMWR Morb Mortal Wkly Rep. 2019;68(3):67-71. doi:10.15585/mmwr.mm6803a3 30677012 PMC6348759

[13] Amsalem D, Yang LH, Jankowski S, Lieff SA, Markowitz JC, Dixon LB. Reducing stigma toward individuals with schizophrenia using a brief video: a randomized controlled trial of young adults. Schizophr Bull. 2021;47(1):7-14. doi:10.1093/schbul/sbaa114 33484269 PMC7825082

[14] Amsalem D, Markowitz JC, Jankowski SE, . Sustained effect of a brief video in reducing public stigma toward individuals with psychosis: a randomized controlled trial of young adults. Am J Psychiatry. 2021;178(7):635-642. doi:10.1176/appi.ajp.2020.20091293 33900809

[15] Amsalem D, Martin A. Reducing depression-related stigma and increasing treatment seeking among adolescents: randomized controlled trial of a brief video intervention. J Child Psychol Psychiatry. Published online April 6, 2021. doi:10.1111/jcpp.13427 33821507

[16] Rowe SL, French RS, Henderson C, Ougrin D, Slade M, Moran P. Help-seeking behaviour and adolescent self-harm: a systematic review. Aust N Z J Psychiatry. 2014;48(12):1083-1095. doi:10.1177/0004867414555718 25335872

[17] Divin N, Harper P, Curran E, Corry D, Leavey G. Help-seeking measures and their use in adolescents: a systematic review. Adolesc Res Rev. 2018;3(1):113-122. doi:10.1007/s40894-017-0078-8

[18] Children Commissioners. Lightning review: access to child and adolescent mental health services. Children Commissioners. 2016. Accessed September 1, 2021. https://www.childrenscommissioner.gov.uk/wp-content/uploads/2017/06/Childrens-Commissioners-Mental-Health-Lightning-Review.pdf

[19] Mehta N, Clement S, Marcus E, . Evidence for effective interventions to reduce mental health-related stigma and discrimination in the medium and long term: systematic review. Br J Psychiatry. 2015;207(5):377-384. doi:10.1192/bjp.bp.114.151944 26527664 PMC4629070

[20] Corrigan PW, Michaels PJ, Vega E, . Key ingredients to contact-based stigma change: a cross-validation. Psychiatr Rehabil J. 2014;37(1):62-64. doi:10.1037/prj0000038 24417232

[21] Corrigan PW, ed. On the Stigma of Mental Illness: Practical Strategies for Research and Social Change. American Psychological Association; 2005. doi:10.1037/10887-000

[22] Chodzen G, Hidalgo MA, Chen D, Garofalo R. Minority stress factors associated with depression and anxiety among transgender and gender-nonconforming youth. J Adolesc Health. 2019;64(4):467-471. doi:10.1016/j.jadohealth.2018.07.006 30241721 PMC6528476

[23] Kosciw JG, Clark CM, Truong NL, Zongrone AD. The 2019 national school climate survey: the experiences of lesbian, gay, bisexual, transgender, and queer youth in our nation’s schools. GLSEN. 2020. Accessed September 1, 2021. https://www.glsen.org/sites/default/files/2021-04/NSCS19-FullReport-032421-Web_0.pdf

[24] Douglass RP, Conlin SE. Minority stress among LGB people: investigating relations among distal and proximal stressors. Curr Psychol. Published online June 27, 2020. doi:10.1007/s12144-020-00885-z

[25] Turban JL, Kraschel KL, Cohen IG. Legislation to criminalize gender-affirming medical care for transgender youth. JAMA. 2021;325(22):2251-2252. doi:10.1001/jama.2021.7764 34028489

[26] Murchison GR, Agénor M, Reisner SL, Watson RJ. School restroom and locker room restrictions and sexual assault risk among transgender youth. Pediatrics. 2019;143(6):e20182902. doi:10.1542/peds.2018-2902 31061223 PMC8849575

[27] Thornicroft G, Mehta N, Clement S, . Evidence for effective interventions to reduce mental-health-related stigma and discrimination. Lancet. 2016;387(10023):1123-1132. doi:10.1016/S0140-6736(15)00298-6 26410341

[28] Koike S, Yamaguchi S, Ojio Y, . A randomised controlled trial of repeated filmed social contact on reducing mental illness-related stigma in young adults. Epidemiol Psychiatr Sci. 2018;27(2):199-208. doi:10.1017/S2045796016001050 27989255 PMC7032789

[29] Clement S, van Nieuwenhuizen A, Kassam A, . Filmed v. live social contact interventions to reduce stigma: randomised controlled trial. Br J Psychiatry. 2012;201(1):57-64. doi:10.1192/bjp.bp.111.093120 22157800

[30] Tadlock BL, Flores AR, Haider-Markel DP, Lewis DC, Miller PR, Taylor JK. Testing contact theory and attitudes on transgender rights. Public Opin Q. 2017;81(4):956-972. doi:10.1093/poq/nfx021

[31] Earle M, Hoffarth MR, Prusaczyk E, MacInnis C, Hodson G. A multilevel analysis of LGBT (lesbian, gay, bisexual, transgender) rights support across 77 countries: the role of contact and country laws. Br J Soc Psychol. 2021;60(3):851-869. doi:10.1111/bjso.12436 33372304

[32] Worthen MGF, Tanzilli A, Caristo C, Lingiardi V. Social contact, social distancing, and attitudes toward LGT individuals: a cross-cultural study of college students in the United States, Italy, and Spain. J Homosex. 2019;66(13):1882-1908. doi:10.1080/00918369.2018.1519302 30346910

[33] Greenburg J, Gaia AC. Interpersonal contact, stereotype acceptance, gender role beliefs, causal attribution, and religiosity as predictors of attitudes toward transgender individuals. Psi Chi J Psychol Res. 2019;24(1):18-32. doi:10.24839/2325-7342.JN24.1.18

[34] Walch SE, Sinkkanen KA, Swain EM, Francisco J, Breaux CA, Sjoberg MD. Using intergroup contact theory to reduce stigma against transgender individuals: impact of a transgender speaker panel presentation: using intergroup contact theory to reduce stigma. J Appl Soc Psychol. 2012;42(10):2583-2605. doi:10.1111/j.1559-1816.2012.00955.x

[35] Chandler J, Rosenzweig C, Moss AJ, Robinson J, Litman L. Online panels in social science research: expanding sampling methods beyond Mechanical Turk. Behav Res Methods. 2019;51(5):2022-2038. doi:10.3758/s13428-019-01273-7 31512174 PMC6797699

[36] Billard TJ. Attitudes toward transgender men and women: development and validation of a new measure. Front Psychol. 2018;9:387. doi:10.3389/fpsyg.2018.00387 29666595 PMC5891633

[37] Norton AT, Herek GM. Heterosexuals’ attitudes toward transgender people: findings from a national probability sample of US adults. Sex Roles. 2013;68(11-12):738-753. doi:10.1007/s11199-011-0110-6

[38] Griffiths KM, Christensen H, Jorm AF, Evans K, Groves C. Effect of web-based depression literacy and cognitive-behavioural therapy interventions on stigmatising attitudes to depression: randomised controlled trial. Br J Psychiatry. 2004;185:342-349. doi:10.1192/bjp.185.4.342 15458995

[39] Wilson CJ, Deane FP, Marshall KL, Dalley A. Reducing adolescents’ perceived barriers to treatment and increasing help-seeking intentions: effects of classroom presentations by general practitioners. J Youth Adolesc. 2008;37(10):1257-1269. doi:10.1007/s10964-007-9225-z

[40] Reinke RR, Corrigan PW, Leonhard C, Lundin RK, Kubiak MA. Examining two aspects of contact on the stigma of mental illness. J Soc Clin Psychol. 2004;23(3):377–89. doi:10.1521/jscp.23.3.377.35457

[41] Chaudoir SR, Wang K, Pachankis JE. What reduces sexual minority stress? a review of the intervention “toolkit”. J Soc Issues. 2017;73(3):586-617. doi:10.1111/josi.12233 29170566 PMC5695701

[42] Parker CM, Hirsch JS, Philbin MM, Parker RG. The urgent need for research and interventions to address family-based stigma and discrimination against lesbian, gay, bisexual, transgender, and queer youth. J Adolesc Health. 2018;63(4):383-393. doi:10.1016/j.jadohealth.2018.05.018 30146436 PMC6344929

[43] Coulter RW, Sang JM, Louth-Marquez W, . Pilot testing the feasibility of a game intervention aimed at improving help seeking and coping among sexual and gender minority youth: protocol for a randomized controlled trial. JMIR Res Protoc. 2019;8(2):e12164. doi:10.2196/12164 30767903 PMC6416896

[44] Huebner DM, Rullo JE, Thoma BC, McGarrity LA, Mackenzie J. Piloting Lead with Love: a film-based intervention to improve parents’ responses to their lesbian, gay, and bisexual children. J Prim Prev. 2013;34(5):359-369. doi:10.1007/s10935-013-0319-y 23943135 PMC3797526

[45] McCarthy J. Mixed views among americans on transgender issues. Gallup. 2021. Accessed September 1, 2021. https://news.gallup.com/poll/350174/mixed-views-among-americans-transgender-issues.aspx

[46] King WM, Hughto JMW, Operario D. Transgender stigma: a critical scoping review of definitions, domains, and measures used in empirical research. Soc Sci Med. 2020;250:112867. doi:10.1016/j.socscimed.2020.112867 32163820 PMC7442603

[47] Cheung RYM, Mak WWS, Tsang PS, Lau JTF. Stigma of psychosis: do diagnostic label, symptom manifestation, and gender matter? Am J Orthopsychiatry. 2018;88(5):529-537. doi:10.1037/ort0000315 29952590

[48] Amsalem D, Lazarov A, Markowitz JC, Smith TE, Dixon LB, Neria Y. Video intervention to increase treatment-seeking by healthcare workers during the COVID-19 pandemic: randomised controlled trial. Br J Psychiatry. 2022;220(1):14-20. doi:10.1192/bjp.2021.54 35045900

[49] Monahan JL, Murphy ST, Zajonc RB. Subliminal mere exposure: specific, general, and diffuse effects. Psychol Sci. 2000;11(6):462-466. doi:10.1111/1467-9280.00289 11202490

[50] Moreland RL, Topolinski S. The mere exposure phenomenon: a lingering melody by Robert Zajonc. Emotion Review. 2010;2(4):329-339. doi:10.1177/1754073910375479

[51] Turban JL, Winer J, Boulware S, VanDeusen T, Encandela J. Knowledge and attitudes toward transgender health. Clin Teach. 2018;15(3):203-207. doi:10.1111/tct.12738 29178596

[52] Webb TL, Sheeran P. Does changing behavioral intentions engender behavior change? a meta-analysis of the experimental evidence. Psychol Bull. 2006;132(2):249-268. doi:10.1037/0033-2909.132.2.249 16536643

