# Supplementary material for: Effect of a Brief Social Contact Video on Transphobia and Depression-Related Stigma Among Adolescents: A Randomized Clinical Trial
Source: JAMA Netw Open. 2022 Feb 25;5(2):e220376. doi: 10.1001/jamanetworkopen.2022.0376 (PMC8881766; doi:10.1001/jamanetworkopen.2022.0376)
Supplement: Supplement 1. — Trial Protocol [file jamanetwopen-e220376-s001.pdf]

Yale

Human Research Protection Program
Institutional Review Boards
FWA00002571
25 Science Park – 3rd Fl., 150 Munson St.
New Haven CT 06520-8327

Telephone: 203-785-4688
http://www.yale.edu/hrpp

November 30, 2021

APPROVAL OF SUBMISSION VIA EXPEDITED REVIEW

Approval Date: 11/30/2021

**Investigator:**	Andres Martin
**Type of Review:**	Modification / Update
**Title of Study:**	Reducing depression-related stigma and increasing help-seeking intentions among adolescents: a randomized controlled trial
**IRB Protocol ID:**	2000028980
**Submission ID:**	MOD00046851

Research activities associated with this submission are approved and may begin consistent with the terms of IRB approval.

The modification request includes: revising the primary endpoint of the Youth trans study only, increasing target enrollment to 500 subjects for each intervention arm for an overall sample size of 1500 and revising the statistical design section.

The IRB finds the modification does not affect subjects' rights or welfare or change subjects' willingness to participate in the study, therefore, reconsenting of subjects is not required.

See the next pages for important reminders and the list of IRB approved documents.

IMPORTANT REMINDERS:

- This research does not require IRB continuing review.

- You are obligated to submit the following to the IRB:

  - **Modifications**: Changes must be submitted with a modification and approved by the IRB prior to implementation except to eliminate immediate hazards to participants. This includes changes to study procedures, informed consent documents, recruitment activities or study personnel.

  - **Reportable New Information:** Information that requires prompt reporting to the IRB must be done so within 5 days of the PI becoming aware of the event (see Policy 710: Reporting Unanticipated Problems Involving Risks to Subjects or Others, including Adverse Events). This includes potential serious noncompliance, continuing noncompliance, and unanticipated problems to subjects or others.

  - **Closure request** (to end the IRB's oversight) when:
    i. The protocol is permanently closed to enrollment,
    ii. All subjects have completed all protocol related interventions and interactions, and
    iii. Analysis of private identifiable information is completed.

- In conducting this activity, you should refer to and follow the Investigator Manual (HRP-103) as applicable, which can be found in the IRB Library within the IRB system.

IRB APROVED DOCUMENTATION:

• Amended protocol - TRACKED CHANGES, Category: IRB Protocol;
• Trans RCT Clinical Trials.pdf, Category: Other;

Please keep this letter with your copy of the approved protocol documents.

ClinicalTrials.gov PRS DRAFT Receipt (Working Version)
Last Update: 08/23/2021 15:59

ClinicalTrials.gov ID: NCT04969003

Study Identification

Unique Protocol ID: 2000028980_a

Brief Title: Reducing Transphobia Among Adolescents Through a Brief Video Intervention

Official Title: Reducing Transphobia Among Adolescents Through a Brief Video Intervention

Secondary IDs:

Study Status

Record Verification: August 2021

Overall Status: Recruiting

Study Start: January 1, 2022 [Anticipated]
NOTE : Study Start Date should be updated and changed to Actual once the first participant is enrolled.

Primary Completion: April 1, 2022 [Anticipated]

Study Completion: July 1, 2022 [Anticipated]

Sponsor/Collaborators

Sponsor: Yale University

Responsible Party: Sponsor

Collaborators:

Oversight

U.S. FDA-regulated Drug: No

U.S. FDA-regulated Device: No

U.S. FDA IND/IDE: No

Human Subjects Review: Board Status: Approved
Approval Number: IRB#2000028980, MOD00040747
Board Name: Yale University Institutional Review Board
Board Affiliation: Yale University
Phone:
Email: HRPP@yale.edu
Address:

P.O. Box 208
New Haven, CT 06520-8327

Data Monitoring: Yes

FDA Regulated Intervention: No

Study Description

Brief Summary: The purposes of this new study are to test among adolescent viewers the utility of brief video-based interventions to:

1. reduce transphobia;
2. reduce depression-related stigma and increase likelihood of treatment-seeking; and
3. examine the role of viewer's sex (male / female / non-binary), race (Black vs non-Black), and sexual orientation (straight vs LGBQ) as independent factors on the outcomes of interest.

Detailed Description: Intervention videos will each be of ~100-second duration and feature four underage professionals (ages 16) acting as simulated patients. All videos will focus on an empowered presenter with depression sharing their personal story regarding depression. They will describe how social supports from family, friends, and community, as well as professional help assisted them in overcoming symptoms of the illness, as well as how being transgender may have had an impact on it. The four actors will include two males (one trans, one cis) and tow females (one trans, one cis).

Assessments will occur at baseline and post-intervention, and will include:

1. Demographics (baseline only);
2. Primary outcome:

   a. Attitudes toward Transgender Men and Women (ATTMW) scale.
3. Secondary outcome:

   a. Transgender attitudes: feelings thermometer, modeled after Norton et al 2013.
   b. Depression-related stigma (Depression Stigma Scale [DSS]);and
   c. Help-seeking (General Help-Seeking Questionnaire [GHSQ]).

For each of study we intend to randomly assign ~1,000 individuals aged 14-18 as follows:

Randomized, in equal proportions, and stratified by sex and race, to view one of four ~100-second videos:

a. Transgender male;
b. Cis-gender male);
c. Transgender female;
d. Cis-gender female.

Conditions

Conditions: Transgenderism
Depression

Keywords:

Study Design

Study Type: Interventional

Primary Purpose: Prevention

Study Phase:	N/A
Interventional Study Model:	Parallel Assignment
Number of Arms:	4
Masking:	Single (Investigator)
Allocation:	Randomized
Enrollment:	1000 [Anticipated]

Arms and Interventions

Arms	Assigned Interventions
Active Comparator: Transgender male (TM)<br>~90-second video of depressed transgender male	Behavioral: Short videos<br>Intervention videos will each be of 90-second duration and feature four underage professionals (ages 16) acting as simulated patients. All videos will focus on an empowered presenter with depression sharing their personal story regarding depression and describe how social supports from family, friends, and community, as well as professional help assisted them in overcoming symptoms of their illness. The actors will include a transgender male, a cis-gender male, a transgender female, and a cis-gender female.
Active Comparator: Cis-gender male (CM)<br>~90-second video of depressed cis-gender male	Behavioral: Short videos<br>Intervention videos will each be of 90-second duration and feature four underage professionals (ages 16) acting as simulated patients. All videos will focus on an empowered presenter with depression sharing their personal story regarding depression and describe how social supports from family, friends, and community, as well as professional help assisted them in overcoming symptoms of their illness. The actors will include a transgender male, a cis-gender male, a transgender female, and a cis-gender female.
Active Comparator: Transgender female (TF)<br>~90-second video of depressed transgender female	Behavioral: Short videos<br>Intervention videos will each be of 90-second duration and feature four underage professionals (ages 16) acting as simulated patients. All videos will focus on an empowered presenter with depression sharing their personal story regarding depression and describe how social supports from family, friends, and community, as well as professional help assisted them in overcoming symptoms of their illness. The actors will include a transgender male, a cis-gender male, a transgender female, and a cis-gender female.
Active Comparator: Cis-gender female (CF)<br>~90-second video of depressed cis-gender female	Behavioral: Short videos<br>Intervention videos will each be of 90-second duration and feature four underage professionals (ages 16) acting as simulated patients. All videos will focus on an empowered presenter with depression sharing their personal story regarding depression and describe how social supports from family, friends, and community, as well as professional help assisted them in overcoming symptoms of their illness. The actors will include a transgender male, a cis-gender male, a transgender female, and a cis-gender female.

Outcome Measures

Primary Outcome Measure:

1. Attitudes toward Transgender Men and Women (ATTMW) scale
   Attitudes toward Transgender Men and Women (ATTMW; Billard, 2018) is a scale to measure transphobic attitudes. It is a 24-item scale consisting of two similar but non-identical 12-item subscales, one for transgender males (ATTM), and one for women (ATTW). In this study, the study team will assign only one of the two subscales, according to the condition that subjects as randomly assigned to: those viewing the transgender adolescent male will be assigned to the ATTM; those to the female, to the ATTW. The results of both subscales are compatible with each other and can be merged into a single ATMW score. Each of the items is a statement on which participants are asked to rate their agreement on a 7-point Likert-type scale: from 1 (strongly disagree) to 7 (strongly agree). Higher scores indicate greater anti-transgender prejudice, while lower scores indicate less prejudice.

   The first 9 items are the same for the ATTM and AATW. Items 10-12 are unique for each of the two scales. These

   [Time Frame: Before / after viewing videos (within 10 minutes)]

Secondary Outcome Measure:

2. Change in transgender attitudes: feelings thermometer
   This study will utilize a scale called feelings thermometer, modeled after Norton and Herek, 2013:

   “Using a scale from zero to 100, please tell us your personal feelings toward each of the following groups of friends, teachers, or colleagues. As you do this task, think of an imaginary thermometer. The warmer or more favorable you feel toward the group, the higher the number you should give it. The colder or less favorable you feel, the lower the number. If you feel neither warm nor cold toward the group, rate it 50.” To familiarize respondents with the response format, they will be first presented with thermometers for “Men in general” and “Women in general,” with each respondent rating her or his own sex first. Next, they rate different gender groups (transgender males, transgender females). Higher ratings (maximum 100) indicate warmer, more favorable feelings toward the target whereas lower ratings (minimum 0) indicate colder, more negative feelings.

   [Time Frame: Before / after viewing videos (within 10 minutes)]

3. Change in Depression-related stigma (Depression Stigma Scale [DSS]; Personal component only): TOTAL SCORE
   The DSS (Christensen, Jorm, Evans, & Groves, 2004) is a self-report instrument composed of two 9-item subscales. The first subscale measures the participants’ own/ personal attitudes, and the second measures participants’ beliefs about the attitudes of others (‘Depression is sign of weakness’ vs. ‘Most people believe that depression is a sign of weakness’). The Personal subscale (DSS-Personal) will be used in this study. The DSS has a 5-point Likert scale ranging from strongly disagree (1) to strongly agree (5). The total score comprises the sum of its item scores, and a higher score indicates more stigma (worse outcome). The DSS-Personal subscale has shown adequate psychometric properties: 0.71 test–retest reliability, 0.76 internal consistency (Griffiths et al., 2004). In our earlier study (Amsalem and Martin, 2021), Cronbach’s a was .83.

   [Time Frame: Before / after viewing videos (within 10 minutes)]

4. Change in Help-seeking (General Help-Seeking Questionnaire [GHSQ]; Emotional and Suicide components): MEAN SCORE
   The General Help-Seeking Questionnaire (GHSQ) (Wilson, Deane, Marshall, & Dalley, 2008) was developed to measure help-seeking intentions from different sources (friend, parent, mental health professional, and others) and is divided into personal-emotional problems and suicidal thoughts (Ibrahim et al., 2019). The instrument consists of 10 items for each part, each rated on a 0 (extremely unlikely) to 7 (extremely likely)Likert scale. Higher scores on this scale indicate more help seeking (better outcome).

   General stem:

   If you were having a personal or emotional problem, how likely is it that you would seek help from the following people?

   [Time Frame: Before / after viewing videos (within 10 minutes)]

5. Change in DSS Item 1
   DDS Item #1: People with depression could snap out of it if they wanted.

   Scored on a 5-point Likert scale ranging from strongly disagree (1) to strongly agree (5)

[Time Frame: Before / after viewing videos (within 10 minutes)]

6. Change in DSS Item 2
   DDS Item #2: Depression is a sign of personal weakness

   Scored on a 5-point Likert scale ranging from strongly disagree (1) to strongly agree (5). A higher score indicates more stigma (worse outcome).

   [Time Frame: Before / after viewing videos (within 10 minutes)]

7. Change in DSS Item 3
   DDS Item #3: Depression is not a real medical illness

   Scored on a 5-point Likert scale ranging from strongly disagree (1) to strongly agree (5). A higher score indicates more stigma (worse outcome).

   [Time Frame: Before / after viewing videos (within 10 minutes)]

8. Change in DSS Item 4
   DDS Item #4: People with depression are dangerous

   Scored on a 5-point Likert scale ranging from strongly disagree (1) to strongly agree (5). A higher score indicates more stigma (worse outcome).

   [Time Frame: Before / after viewing videos (within 10 minutes)]

9. Change in DSS Item 5
   DDS Item #5: It is best to avoid people with depression, so you don’t become depressed yourself

   Scored on a 5-point Likert scale ranging from strongly disagree (1) to strongly agree (5). A higher score indicates more stigma (worse outcome).

   [Time Frame: Before / after viewing videos (within 10 minutes)]

10. Change in DSS Item 6
    DDS Item #6: People with depression are unpredictable

    Scored on a 5-point Likert scale ranging from strongly disagree (1) to strongly agree (5). A higher score indicates more stigma (worse outcome).

    [Time Frame: Before / after viewing videos (within 10 minutes)]

11. Change in DSS Item 7
    DDS Item #7: If I had depression, I would not tell anyone

    Scored on a 5-point Likert scale ranging from strongly disagree (1) to strongly agree (5). A higher score indicates more stigma (worse outcome).

    [Time Frame: Before / after viewing videos (within 10 minutes)]

12. Change in DSS Item 8
    DDS Item #8 : I would not employ someone if I knew they had been depressed

    Scored on a 5-point Likert scale ranging from strongly disagree (1) to strongly agree (5). A higher score indicates more stigma (worse outcome).

    [Time Frame: Before / after viewing videos (within 10 minutes)]

13. Change in DSS Item 9
    DDS Item #9: I would not vote for a politician if I knew they had been depressed

    Scored on a 5-point Likert scale ranging from strongly disagree (1) to strongly agree (5). A higher score indicates more stigma (worse outcome).

    [Time Frame: Before / after viewing videos (within 10 minutes)]

14. Change in GHSQ Emotional Item 1
    If you were having a personal or emotional problem, how likely is it that you would seek help from the following people?

    Intimate partner (e.g., girlfriend, boyfriend)

Measured with a 7-point Likert scale ranging from 1 (extremely unlikely) to 7 (extremely likely). Higher scores on this item indicate more help seeking (better outcome).

[Time Frame: Before / after viewing videos (within 10 minutes)]

15. Change in GHSQ Emotional Item 2
    If you were having a personal or emotional problem, how likely is it that you would seek help from the following people?

    Friend (not related to you)

    Measured with a 7-point Likert scale ranging from 1 (extremely unlikely) to 7 (extremely likely). Higher scores on this item indicate more help seeking (better outcome).

    [Time Frame: Before / after viewing videos (within 10 minutes)]

16. Change in GHSQ Emotional Item 3
    If you were having a personal or emotional problem, how likely is it that you would seek help from the following people?

    Parent

    Measured with a 7-point Likert scale ranging from 1 (extremely unlikely) to 7 (extremely likely). Higher scores on this item indicate more help seeking (better outcome).

    [Time Frame: Before / after viewing videos (within 10 minutes)]

17. Change in GHSQ Emotional Item 4
    If you were having a personal or emotional problem, how likely is it that you would seek help from the following people?

    Other relative/family member

    Measured with a 7-point Likert scale ranging from 1 (extremely unlikely) to 7 (extremely likely). Higher scores on this item indicate more help seeking (better outcome).

    [Time Frame: Before / after viewing videos (within 10 minutes)]

18. Change in GHSQ Emotional Item 5
    If you were having a personal or emotional problem, how likely is it that you would seek help from the following people?

    Mental health professional (e.g., psychologist, social worker, counselor)

    Measured with a 7-point Likert scale ranging from 1 (extremely unlikely) to 7 (extremely likely). Higher scores on this item indicate more help seeking (better outcome).

    [Time Frame: Before / after viewing videos (within 10 minutes)]

19. Change in GHSQ Emotional Item 6
    If you were having a personal or emotional problem, how likely is it that you would seek help from the following people?

    Phone helpline (e.g., lifeline)

    Measured with a 7-point Likert scale ranging from 1 (extremely unlikely) to 7 (extremely likely). Higher scores on this item indicate more help seeking (better outcome).

    [Time Frame: Before / after viewing videos (within 10 minutes)]

20. Change in GHSQ Emotional Item 7
    If you were having a personal or emotional problem, how likely is it that you would seek help from the following people?

    Doctor/GP

    Measured with a 7-point Likert scale ranging from 1 (extremely unlikely) to 7 (extremely likely). Higher scores on this item indicate more help seeking (better outcome).

    [Time Frame: Before / after viewing videos (within 10 minutes)]

21. Change in GHSQ Emotional Item 8
    If you were having a personal or emotional problem, how likely is it that you would seek help from the following people?

    Minister or religious leader (e.g., Priest, Rabbi, Chaplain)

    Measured with a 7-point Likert scale ranging from 1 (extremely unlikely) to 7 (extremely likely). Higher scores on this item indicate more help seeking (better outcome).

    [Time Frame: Before / after viewing videos (within 10 minutes)]
22. Change in GHSQ Emotional Item 9
    If you were having a personal or emotional problem, how likely is it that you would seek help from the following people?

    I would not seek help from anyone

    REVERSE SCORED ITEM Measured with a 7-point Likert scale ranging from 1 (extremely unlikely) to 7 (extremely likely). LOWER scores on this item indicate more help seeking (better outcome).

    [Time Frame: Before / after viewing videos (within 10 minutes)]
23. Change in GHSQ Emotional Item 10
    If you were having a personal or emotional problem, how likely is it that you would seek help from the following people?

    Emotional Item # 10: I would seek help from another not listed above

    Measured with a 7-point Likert scale ranging from 1 (extremely unlikely) to 7 (extremely likely). Higher scores on this item indicate more help seeking (better outcome).

    [Time Frame: Before / after viewing videos (within 10 minutes)]
24. Change in GHSQ Suicide Item 1
    If you were having suicidal thoughts, how likely is it that you would seek help from the following people?

    Intimate partner (e.g., girlfriend, boyfriend)

    Measured with a 7-point Likert scale ranging from 1 (extremely unlikely) to 7 (extremely likely). Higher scores on this item indicate more help seeking (better outcome).

    [Time Frame: Before / after viewing videos (within 10 minutes)]
25. Change in GHSQ Suicide Item 2
    If you were having suicidal thoughts, how likely is it that you would seek help from the following people?

    Friend (not related to you)

    Measured with a 7-point Likert scale ranging from 1 (extremely unlikely) to 7 (extremely likely). Higher scores on this item indicate more help seeking (better outcome).

    [Time Frame: Before / after viewing videos (within 10 minutes)]
26. Change in GHSQ Suicide Item 3
    If you were having suicidal thoughts, how likely is it that you would seek help from the following people?

    Parent

    Measured with a 7-point Likert scale ranging from 1 (extremely unlikely) to 7 (extremely likely). Higher scores on this item indicate more help seeking (better outcome).

    [Time Frame: Before / after viewing videos (within 10 minutes)]
27. Change in GHSQ Suicide Item 4
    Other relative/family member

    Measured with a 7-point Likert scale ranging from 1 (extremely unlikely) to 7 (extremely likely). Higher scores on this item indicate more help seeking (better outcome).

    [Time Frame: Before / after viewing videos (within 10 minutes)]

28. Change in GHSQ Suicide Item 5
If you were having suicidal thoughts, how likely is it that you would seek help from the following people?

Mental health professional (e.g., psychologist, social worker, counselor)

Measured with a 7-point Likert scale ranging from 1 (extremely unlikely) to 7 (extremely likely). Higher scores on this item indicate more help seeking (better outcome).

[Time Frame: Before / after viewing videos (within 10 minutes)]

29. Change in GHSQ Suicide Item 6
If you were having suicidal thoughts, how likely is it that you would seek help from the following people?

Phone helpline (e.g., lifeline)

Measured with a 7-point Likert scale ranging from 1 (extremely unlikely) to 7 (extremely likely). Higher scores on this item indicate more help seeking (better outcome).

[Time Frame: Before / after viewing videos (within 10 minutes)]

30. Change in GHSQ Suicide Item 7
If you were having suicidal thoughts, how likely is it that you would seek help from the following people?

Doctor/GP

Measured with a 7-point Likert scale ranging from 1 (extremely unlikely) to 7 (extremely likely). Higher scores on this item indicate more help seeking (better outcome).

[Time Frame: Before / after viewing videos (within 10 minutes)]

31. Change in GHSQ Suicide Item 8
If you were having suicidal thoughts, how likely is it that you would seek help from the following people?

Minister or religious leader (e.g., Priest, Rabbi, Chaplain)

Measured with a 7-point Likert scale ranging from 1 (extremely unlikely) to 7 (extremely likely). Higher scores on this item indicate more help seeking (better outcome).

[Time Frame: Before / after viewing videos (within 10 minutes)]

32. Change in GHSQ Suicide Item 9
If you were having suicidal thoughts, how likely is it that you would seek help from the following people?

GHSQ Item# 9: I would not seek help from anyone

REVERSE SCORED ITEM Measured with a 7-point Likert scale ranging from 1 (extremely unlikely) to 7 (extremely likely). LOWER scores on this item indicate more help seeking (better outcome).

[Time Frame: Before / after viewing videos (within 10 minutes)]

33. Change in GHSQ Suicide Item 10
If you were having suicidal thoughts, how likely is it that you would seek help from the following people?

GHSQ Item# 10:I would seek help from another not listed above

Measured with a 7-point Likert scale ranging from 1 (extremely unlikely) to 7 (extremely likely). Higher scores on this item indicate more help seeking (better outcome).

[Time Frame: Before / after viewing videos (within 10 minutes)]

34. Change in ATTMW Item 1
ATTMW Item#1: Transgender men/women will never really be men/women

Score on a 7-point Likert-type scale: from 1 (strongly disagree) to 7 (strongly agree). Higher scores indicate greater anti-transgender prejudice, while lower scores indicate less prejudice.

[Time Frame: Before / after viewing videos (within 10 minutes)]

35. Change in ATTMW Item 2
ATTMW Item#2:Transgender men/women are not really men/women

Score on a 7-point Likert-type scale: from 1 (strongly disagree) to 7 (strongly agree). Higher scores indicate greater anti-transgender prejudice, while lower scores indicate less prejudice.

[Time Frame: Before / after viewing videos (within 10 minutes)]

36. Change in ATTMW Item 3
    ATTMW Item#3:Transgender men/women are only able to look like men/women, but not be men/women

    Score on a 7-point Likert-type scale: from 1 (strongly disagree) to 7 (strongly agree). Higher scores indicate greater anti-transgender prejudice, while lower scores indicate less prejudice.

    [Time Frame: Before / after viewing videos (within 10 minutes)]

37. Change in ATTMW Item 4
    ATTMW Item#4:Transgender men/women are unable to accept who they really are

    Score on a 7-point Likert-type scale: from 1 (strongly disagree) to 7 (strongly agree). Higher scores indicate greater anti-transgender prejudice, while lower scores indicate less prejudice.

    [Time Frame: Before / after viewing videos (within 10 minutes)]

38. Change in ATTMW Item 5
    ATTMW Item#5:Transgender men/women are trying to be someone they're not

    Score on a 7-point Likert-type scale: from 1 (strongly disagree) to 7 (strongly agree). Higher scores indicate greater anti-transgender prejudice, while lower scores indicate less prejudice.

    [Time Frame: Before / after viewing videos (within 10 minutes)]

39. Change in ATTMW Item 6
    Transgender adolescent men/women are denying their DNA

    Score on a 7-point Likert-type scale: from 1 (strongly disagree) to 7 (strongly agree). Higher scores indicate greater anti-transgender prejudice, while lower scores indicate less prejudice.

    [Time Frame: Before / after viewing videos (within 10 minutes)]

40. Change in ATTMW Item 7
    ATTMW Item #8:Transgender men/women cannot just "identify" as men/women

    Score on a 7-point Likert-type scale: from 1 (strongly disagree) to 7 (strongly agree). Higher scores indicate greater anti-transgender prejudice, while lower scores indicate less prejudice.

    [Time Frame: Before / after viewing videos (within 10 minutes)]

41. Change in **ATTM** Item 11
    ATTM Item #11: Transgender men are misguided

    Score on a 7-point Likert-type scale: from 1 (strongly disagree) to 7 (strongly agree). Higher scores indicate greater anti-transgender prejudice, while lower scores indicate less prejudice.

    [Time Frame: Before / after viewing videos (within 10 minutes)]

42. Change in ATTMW Item 8
    ATTMW Item #10:Transgender men/women are unnatural

    Score on a 7-point Likert-type scale: from 1 (strongly disagree) to 7 (strongly agree). Higher scores indicate greater anti-transgender prejudice, while lower scores indicate less prejudice.

    [Time Frame: Before / after viewing videos (within 10 minutes)]

43. Change in ATTMW Item 9
    ATTMW Item #11:Transgender men/women don't really understand what it means to be a man/woman

    Score on a 7-point Likert-type scale: from 1 (strongly disagree) to 7 (strongly agree). Higher scores indicate greater anti-transgender prejudice, while lower scores indicate less prejudice.

    [Time Frame: Before / after viewing videos (within 10 minutes)]

44. Change in **ATTM** Item 11
    ATTMW Item #11:Transgender men are emotionally unstable

Score on a 7-point Likert-type scale: from 1 (strongly disagree) to 7 (strongly agree). Higher scores indicate greater anti-transgender prejudice, while lower scores indicate less prejudice.

[Time Frame: Before / after viewing videos (within 10 minutes)]

45. Change in **ATTW** Item 11
Transgender women are defying their nature.

[Time Frame: Before / after viewing videos (within 10 minutes)]

46. Change in **ATTM** Item 10
Transgender men seem absolutely normal to me (R)

(R) = REVERSE-SCORED

Scored on a 7-point Likert-type scale: from 1 (strongly disagree) to 7 (strongly agree). Higher scores indicate greater anti-transgender prejudice, while lower scores indicate less prejudice.

[Time Frame: Before / after viewing videos (within 10 minutes)]

47. Change in **ATTW** Item 10
Transgender women only think they are females

Scored on a 7-point Likert-type scale: from 1 (strongly disagree) to 7 (strongly agree). Higher scores indicate greater anti-transgender prejudice, while lower scores indicate less prejudice.

[Time Frame: Before / after viewing videos (within 10 minutes)]

48. Change in **ATTW** Item 12
There is something unique about being a woman that transgender adolescent females can never experience

Scored on a 7-point Likert-type scale: from 1 (strongly disagree) to 7 (strongly agree). Higher scores indicate greater anti-transgender prejudice, while lower scores indicate less prejudice.

[Time Frame: Before / after viewing videos (within 10 minutes)]

NOTE : More than one Outcome Measure has the Title 'Change in **ATTM** Item 11' and the Time Frame 'Before / after viewing videos (within 10 minutes)'

Eligibility

Minimum Age: 14 Years

Maximum Age: 18 Years

Sex: All

Gender Based: No

Accepts Healthy Volunteers: Yes

Criteria: Inclusion Criteria:

- English-speaking
- Living in the US
- Ages 14 - 18

Exclusion Criteria:

- None

Contacts/Locations

Central Contact Person: Andres Martin, MD, MPH
Telephone: 2034942355
Email: andres.martin@yale.edu

Central Contact Backup:

Study Officials: NOTE : Study Official is required by the WHO and ICMJE.

Locations: **United States, Connecticut**
Yale Child Study Center
[Not yet recruiting]
New Haven, Connecticut, United States, 06510
Contact: Andres Martin, MD, MPH 203-494-2355 andres.martin@yale.edu

Yale Child Center
[Recruiting]
New Haven, Connecticut, United States, 06510
Contact: Andres Martin, MD 203-494-2355 andres.martin@yale.edu
Principal Investigator: Andres Martin, MD

IPDSharing

Plan to Share IPD: No

References

Citations:

Griffiths KM, Christensen H, Jorm AF, Evans K, Groves C. Effect of web-based depression literacy and cognitive-behavioural therapy interventions on stigmatising attitudes to depression: randomised controlled trial. Br J Psychiatry. 2004 Oct;185:342-9. PubMed 15458995

Wilson CJ, Deane FP, Marshall KL, Dalley A. Adolescents' suicidal thinking and reluctance to consult general medical practitioners. J Youth Adolesc. 2010 Apr;39(4):343-56. doi: 10.1007/s10964-009-9436-6. Epub 2009 Jul 15. PubMed 20229227

Norton AT, Herek GM. Heterosexuals' attitudes toward transgender people: findings from a national probability sample of US Adults. Sex Roles. 2013;68(11-12):738-753. doi:10.1007/s11199-011-0110-6

Billard TJ. Attitudes Toward Transgender Men and Women: Development and Validation of a New Measure. Front Psychol. 2018 Apr 3;9:387. doi: 10.3389/fpsyg.2018.00387. eCollection 2018. PubMed 29666595

Links:

Available IPD/Information:

U.S. National Library of Medicine | U.S. National Institutes of Health | U.S. Department of Health & Human Services
