# Supplementary material for: Effect of a Brief Social Contact Video on Transphobia and Depression-Related Stigma Among Adolescents: A Randomized Clinical Trial
Source: JAMA Netw Open. 2022 Feb 25;5(2):e220376. doi: 10.1001/jamanetworkopen.2022.0376 (PMC8881766; doi:10.1001/jamanetworkopen.2022.0376)
Supplement: Supplement 2. — eAppendix 1. Links to Four Video Conditions eAppendix 2. Comparison Between Baseline and Postintervention Scores (n = 1,009) on the General Help-Seeking Questionnaire (GHSQ) [file jamanetwopen-e220376-s002.pdf]

Supplementary Online Content

Amsalem D, Halloran J, Penque B, Celentano J, Martin A. Effect of a brief social contact video on transphobia and depression-related stigma among adolescents: a randomized clinical trial. *JAMA Netw Open*. 2022;5(2):e220376. doi:10.1001/jamanetworkopen.2022.0376

eAppendix 1. Links to Four Video Conditions

eAppendix 2. Comparison Between Baseline and Postintervention Scores (n = 1,009) on the

General Help-Seeking Questionnaire (GHSQ)

This supplementary material has been provided by the authors to give readers additional information about their work.

© 2022 Amsalem D et al. *JAMA Network Open.*

eAppendix 1. Links to Four Video Conditions

(Adolescents and their parents provided permission)

1. Transgender adolescent female (TF) https://youtu.be/aP4hfpUs8G8
2. Transgender adolescent male (TM) https://youtu.be/SLbsuMVbUS4
3. Cisgender adolescent female (CF) https://youtu.be/Np06IRk3WoU
4. Cisgender adolescent male (CM) https://youtu.be/lNcynLyfRLQ

© 2022 Amsalem D et al. *JAMA Network Open.*

eAppendix 2. Comparison Between Baseline and Postintervention Scores (n = 1,009) on the General Help-Seeking Questionnaire (GHSQ)

If you were having a personal or emotional problem, how likely is it that you would seek help from the following people?		Baseline	Post-intervention	Statistic	*p*
1	Intimate partner (e.g., girlfriend, boyfriend)	5.4 (1.8)	5.5 (1.8)	NS	-
2	Friend (not related to you)	4.9 (1.7)	5.0 (1.7)	NS	-
3	**Parent**	**3.9 (1.7)**	**4.1 (2.1)**	**4.9**	**<0.001**
4	**Other family member**	**3.3 (1.9)**	**3.4 (2.0)**	**3.2**	**<0.001**
5	Mental health professional (e.g., psychologist, social worker, counselor)	4.5 (2.0)	4.5 (2.1)	NS	-
6	Phone helpline (e.g., lifeline)	3.0 (1.9)	3.0 (2.0)	NS	-
7	**Doctor/General practitioner**	**3.6 (2.0)**	**3.8 (2.1)**	**3.6**	**<0.001**
8	Minister or religious leader (e.g., Priest, Rabbi, Chaplain)	2.6 (2.0)	2.6 (2.2)	NS	-
9	**I would not seek help from anyone**	**4.7 (2.1)**	**5.0 (2.0)**	**4.2**	**<0.001**
10	I would seek help from another not listed above	2.8 (2.0)	2.8 (2.0)	NS	-

Note: GHSQ scores are reported as mean(±SD); bold indicates significance at $p<0.001$ (paired t-test). Items ranged from 1 (Extremely unlikely) to 7 (Extremely likely) on a Likert-type scale, with higher scores indicating more help-seeking; NS, non-significant; item #9 is reverse-scored.

© 2022 Amsalem D et al. *JAMA Network Open.*
