# Supplementary material for: Effect of a Brief Social Contact Video on Transphobia and Depression-Related Stigma Among Adolescents: A Randomized Clinical Trial
Source: JAMA Netw Open. 2022 Feb 25;5(2):e220376. doi: 10.1001/jamanetworkopen.2022.0376 (PMC8881766; doi:10.1001/jamanetworkopen.2022.0376)
Supplement: Supplement 3. — Data Sharing Statement [file jamanetwopen-e220376-s003.pdf]

Data Sharing Statement

Amsalem. Effect of a Brief Social Contact Video on Transphobia and Depression-Related Stigma Among Adolescents. *JAMA Netw Open*. Published February 25, 2022. doi:10.1001/jamanetworkopen.2022.0376

Data
Data available: No

Additional Information
Explanation for why data not available: Data will be shared by request
